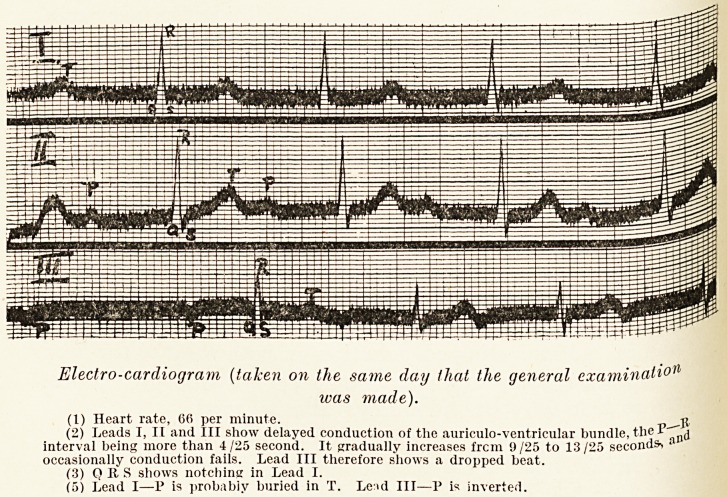# The Evidences of Heart Failure, and the Signs and Symptoms of Three Cases Personally Examined
*A British Medical Association Prize Essay.


**Published:** 1928

**Authors:** P. Christine Vine


### THE EVIDENCES OF HEART FAILURE, AND THE SIGNS AND SYMPTOMS OF THREE CASES PERSONALLY EXAMINED.*

BY

P. CHRISTINE VINE.

---

### INTRODUCTION.

A FEW words may be devoted to the term "heart failure" and its underlying causes by way of introduction. Some explanation is, indeed, necessary, owing to the fact that "heart failure" is a term applied both to a condition in which death occurs immediately, and to one in which symptoms appear in such an insidious manner that the patient scarcely discerns their onset. In spite of the apparent disparity of these two extremes, the underlying principle is the same, *i.e.* the heart muscle has been damaged. When an individual cannot maintain an efficient circulation during the performance of the usual activities of daily life he then suffers from heart failure.

During recent years the whole conception of heart failure has been revolutionised. The old school of thought based their theories on the fact that a valve of the heart had become defective. They maintained this to be the primary cause of the heart failure. They believed that in time all four chambers of the heart became involved, and the prognosis depended on whether compensation was established or not. In the former case hypertrophy of the heart muscle occurred, but in the latter dilatation. The new school holds that

* A British Medical Association Prize Essay.

heart failure is due to injury to the muscle wall of the heart. Usually the extent of the damage is such that a valve is involved, though this does not necessarily occur. If the muscle is damaged to any considerable extent, heart failure will become evident even if all the valves are intact. This damage is considered to be the primary cause of dilatation.

The causes of injury to the heart muscle may be classified under three headings :—

1. *Infections*, as in rheumatism, ulcerative endocarditis and syphilis.

2. *Intoxications*, such as thyroid, alcohol and bacterial toxins, *e.g.* pneumococcal and diphtheria toxins.

3. *Degenerations*, such as those caused by high blood pressure and senility.

### CLINICAL EVIDENCES OF CARDIAC FAILURE.

These may be divided into two main groups, as it generally manifests itself in one of two ways :—

1. By the onset of symptoms due to an inefficient flow of blood through the organs of the body. Sometimes this is known as the congestive type.

2. By the occurrence of pain due to exhaustion of the heart muscle. This is known as the anginal type.

Under the first of these headings several signs and symptoms may be considered.

*Dyspnœa.*—This is perhaps one of the most important symptoms, as it is one of the earliest manifestations of heart failure, and is rarely absent in any individual suffering from the congestive type of heart failure.

It has been shown by Peabody, Meyer and Du Bois[1] that patients suffering from cardiac dyspnœa have a slightly increased metabolic demand for the intake of oxygen. This, however, is not sufficient to cause breathlessness during rest. The chief cause is the inability on the part of the heart muscle to pump sufficient blood to the medulla; another result of the same inability is a deficient oxygenation of the blood, and in the same way an acidosis is set up in the capillaries due to excess carbon dioxide. A second cause, no doubt, exists in the pulmonary congestion, and, if present, pulmonary œdema, both of which reduce the respiratory capacity. These factors, by producing a state of deficient æration of the blood, are liable to cause damage to the myocardium, and in consequence a dangerous vicious circle may be set up.

In order to determine whether shortness of breath on exertion be due to cardiac disease or not, it must be compared with the patient's previous powers of exertion without the production of respiratory distress. One individual may undertake a great deal more exercise than another without becoming short of breath. The greater the damage to the heart muscle the more readily will breathlessness be induced.

*Orthopnœa.*—If an individual suffers from slight dyspnœa on exertion and the condition is rapidly progressive, in due course he will suffer from constant dyspnœa, *i.e.* he will be dyspnœic during rest. This is known as orthopnœa, when the patient is compelled to assume a sitting posture. An attack of orthopnœa may be brought on by prolonged over-exertion in cases exhibiting only a comparatively slight degree of cardiac failure. This fact was illustrated by a case seen at the Bristol General Hospital in June, 1926. The patient was a girl aged 18, who had suffered from rheumatic fever when 3½ years old. For several years she had maintained fairly good health, but during the last six months previous to admission her work had been of a somewhat strenuous nature. During two months of this time she had been a chamber-maid in one of the largest hotels in Bristol. Owing to the fact that she had been carrying heavy loads, she had developed some scoliosis of the spine. On admission the patient stated that she walked several miles only a few days previously. On examination she showed several signs of heart failure, particularly orthopnœa. Her condition greatly improved with rest and treatment. Sometimes sufficient rest will cause orthopnœa to disappear entirely. When orthopnœa occurs in patients who have not been over-exerting themselves it is a grave sign. It may mean that the heart has become arrhythmic ; for instance, it may signify the onset of auricular fibrillation some days or hours previously. In such cases it is usually accompanied by other signs of cardiac failure.

*Cheyne-Stokes Breathing.*—By this is meant a form of periodic respiration in which at regular intervals a period of apnœa (*i.e.* entire absence of respiration) occurs, and may last from 10 to 60 seconds. At the end of this time respiration recommences, but is at first almost imperceptible. The inspirations and expirations gradually become more and more forcible until a maximum is reached, after which they diminish in force until the period of apnœa is reached. When Cheyne-Stokes breathing occurs as a sign of cardiac failure, it is usually of serious import. It signifies myocardial degeneration, and is therefore often seen in senile heart cases. It may occur in combination with auricular fibrillation or with auricular flutter.

It is not necessarily, however, a sign of cardiac failure if it occurs apart from other signs. It may occur in elderly people, living fairly active lives, and is then of little significance. It may also occur in people with a high blood pressure and in cases of advanced arteriosclerosis.

Cheyne-Stokes breathing is most commonly seen whilst the patient is sleeping, but it may occur whilst the patient is sitting quietly in a chair. Its presence should be suspected when the patient complains of being constantly awakened out of his sleep by a feeling of suffocation. The individual may awake in such a terror-stricken state that he jumps out of bed. Sometimes a patient will fall asleep during the apnœic period, but will awake and resume his trend of thought when respirations return.

*Cardiac asthma.*—This is a peculiar form of dyspnœa which is a manifestation of heart failure. It also occurs most frequently during the night. It is characterised by very severe dyspnœa, the onset of which is extremely sudden. The patient awakes out of his sleep with a great sensation of suffocation. He is compelled to sit up, and the breathing is extremely laboured. Respirations may be accompanied by wheezing sounds, and the patient may expectorate a considerable amount of frothy sputum.

This dyspnœa may last as long as half an hour or longer. If it occurs during the night the patient usually prefers to remain propped up in bed. Further sleep is often troubled and disturbed. It may indicate myocardial degeneration, and it frequently occurs in senile heart cases. It is also a manifestation of heart failure following high blood pressure, or in cases in which arterial changes have taken place. Occasionally it is seen in patients in middle life suffering from heart disease. It may be associated with angina pectoris, when it is an exceedingly grave sign.

*Cerebral symptoms.*—Insufficient driving power of the heart, resulting in an inefficient flow of blood to the brain, is sometimes the cause of cerebral symptoms, such as giddiness. This may be of a mild degree or cause the patient to fall. In severe cases there may be complete loss of consciousness. The Adams-Stokes syndrome is sometimes seen, a condition characterised by a paroxysmal infrequency of the pulse associated with epileptiform attacks. This is usually a consequence of auriculo-ventricular heart block, and the blood supply to the brain may be completely arrested. Sometimes the patient exhibits unusually early fatigue after mental effort, or a loss of memory. Hallucinations or insanity may be present, the patient becoming restless, or even violent. This is also sometimes a characteristic of myocardial degeneration of senility.

*Cyanosis.*—The diminished arterial supply, leading to deficient oxygenation of the blood and accumulation of carbon dioxide, results in a bluish flush of the skin, chiefly of the cheeks and extremities, known as cyanosis. Later these parts, the lobes of the ears and the nose, may show lividity. Cyanosis is more frequently seen in cases of mitral disease than aortic, as in the former condition there is a great venous engorgement due to obstruction or regurgitation of blood at the mitral valve.

*Œdema.*—Several factors appear to determine the appearance of this sign of heart failure. It occurs in patients in whom there is a central obstruction to the circulation due to valvular disease of the heart, or to general cardiac weakness. If the patient is up and about this œdema is seen first in the feet and legs, but if the patient is in bed it will be more marked in the lumbar region of the back or in the dependent parts of the lungs. This demonstrates that gravity is one of the factors. In chronic heart disease there is always a long-continued increase of pressure in the capillaries, and in cases in which there is great venous engorgement there is the additional factor of diminished absorption by the veins. Cases have been recorded in which complete obstruction of the inferior vena cava has been found on post-mortem examination, but the patient has shown no signs of œdema. This proves that increased pressure in the capillaries and decreased absorption by the veins are not the only causal factors. Another, probably, is some damage to the capillary wall and surrounding tissues caused by the increased pressure and the malnutrition of the tissues, which is the inevitable result of the inefficient blood supply. It is possible that this injury to the capillary walls, allowing of an increased transudation of lymph, is the main causal factor.

Œdema round the ankles occurs in many healthy individuals after standing for some considerable time. It is also present in certain other diseases, such as nephritis. It must not be taken as a sign of cardiac failure unless other signs of heart disease are present.

*Enlargement of the liver.*—Swelling of the liver in cardiac disease is due to localised œdema. One of the factors in its production is that normally the force of the circulation is least in such organs as this and the kidneys. The commonest cause of chronic venous congestion of the liver is mitral disease. It may also occur as the result of obstruction to the pulmonary circulation. If pulsation of the liver is felt, the enlargement is cardiac in origin. When determining the cause of liver enlargement, an examination of the physical signs of the heart is important, as it should only be considered a sign of cardiac failure when occurring in combination with dilatation of the heart.

*Albuminuria.*—If albumen is found to be present in the urine, an estimation of the amount should always be obtained as by Esbach's test. A considerable amount of albumen may be found in advanced heart failure. Albuminuria is a result of chronic venous congestion of the kidneys; the more severe the congestion the greater is the amount of albumen in the urine.

*Œdema of the lungs.*—Finally, another sign due to a deficient output of blood is œdema of the lungs. The patient suffers at the same time from orthopnœa and, owing to the fact that he has assumed a sitting posture, œdema is usually found at the bases of the lungs. Its presence may be detected by auscultation; in the early stages fine crepitations will be heard during the first deep inspiration. In time, if the condition is progressive, the crepitations become persistent and are heard during every inspiration.

*The anginal form* of heart failure, *i.e.* the second type mentioned under which another group of evidences can be placed, may now be considered. This form of heart failure is characterised by pain, the cause of which is exhaustion of the heart muscle; the output of blood may not be altered to any extent from the normal. In some cases, however, both forms of heart failure are present at the same time.

The *pain* may be a constant full ache in the præcordium, or a recurring sharp pain; a more serious form of pain, however, is that known as angina pectoris. This latter occurs in attacks, and is brought on by effort. Usually the pain is an immediate result of the exertion. The typical pain of angina pectoris is always felt in definite regions, across the left side of the chest (including the region of the sternum), in the left arm, but in rare instances in the right arm; it may also occur at the back of the neck behind the ear and along the jaw. The pain is of a radiating character usually, though it may remain localised in some part of the chest. It most commonly passes from the chest to the left armpit or shoulder (occasionally it passes to both arms), then it travels down the arm on the ulnar side, not usually farther than the elbow, although it may travel as far as the tips of the fingers, affecting the little and ring fingers.

The common causes of angina pectoris are sclerosis of the coronary arteries and syphilis. The associated cardiac condition present may be fibroid myocarditis or an aortic lesion such as an aneurysm, syphilitic aortitis, or aortic incompetence. When angina pectoris occurs in any one of these it is a grave sign, as the condition has usually advanced to an extreme degree before the occurrence of pain. An estimate of the damage to the heart muscle can be obtained by finding out the amount of effort the patient can make under favourable conditions, and by noting the effect of treatment.

Other evidences of the above-named diseases will be found on careful examination of the patient, such as enlargement of the heart. The old school of thought laid a great deal of stress on the differentiation between hypertrophy and dilatation. In practice, however, this differentiation is by no means easy.

Another symptom accompanying or preceding an attack of angina pectoris is a sense of constriction of the chest. Occasionally this is a more distressing symptom to the patient than the pain. It is probably due to a contraction of the intercostal muscles.

*A sense of depression* sometimes accompanies an attack of angina pectoris. The patient has a feeling of impending death. This depression is not pathognomonic of angina pectoris, it sometimes occurs in auricular flutter.

*Vaso-motor disturbances* are discernible in some. This may take the form of pallor or of flushing. Both of these disturbances are followed by profuse perspiration.

*An increased flow of saliva* occurs in some patients during an attack. This is particularly liable to occur if the pain is in the jaw.

*An increased flow of urine* frequently follows an attack, and large quantities may be passed.

### PHYSIOLOGICAL TYPES OF CARDIAC FAILURE, WITH ILLUSTRATIVE CASES.

Various means and mechanical contrivances have been devised whereby certain evidences of heart failure can be detected. For the purpose of dealing with these and their application, heart failure may be divided physiologically into three different groups. The heart is composed of three parts, each having its own peculiar function. They are (*a*) the auricles, in which the contraction wave is originated, and which by their contraction drive the blood into the ventricles ; (*b*) the ventricles, which by their forcible contraction drive the blood through the body ; (*c*) the regulating system, consisting of the sino-auricular node, auriculo-ventricular node and bundle. The three groups then are termed auricular, ventricular and auriculo-ventricular failure. There is no fast line of demarcation between these three types. A patient suffering from auricular failure eventually dies from ventricular failure. On the other hand, a patient suffering from advanced ventricular failure, as in syphilitic aortic incompetence, may suddenly develop an arrhythmia due to auricular fibrillation. Each group, however, has certain distinctive evidences.

*Auricular failure* occurs in its most characteristic form in advanced cardiac rheumatism. It has four main features. The first is total arrhythmia, no trace of regular rhythm remaining. The pulse is usually rapid and very irregular in force and frequency ; the heart beat, however, is even more rapid, as a number of the less forcible contractions fail to reach the wrist.

The second sign is the loss of all evidence of auricular systole. On auscultation no presystolic murmur is heard, and on palpation no presystolic thrill is felt, although these may have been marked features of the case before the onset of auricular failure. If a polygram be taken the jugular curve shows no evidence of an auricular systolic movement. Its absence is also shown by an electro-cardiogram.

By means of the last two methods mentioned, the third sign, *i.e.* auricular fibrillation, can be demonstrated, minute, rapid, irregular movements being recorded.

The fourth sign is the increase in severity of dyspnœa, cyanosis, jugular distension, liver enlargement and crepitations in the lungs observable shortly after the onset of auricular failure.

Case 1.—E. T., female, aged 54. Married. Admitted to the Bristol General Hospital in September, 1925. Died five weeks later.

*Diagnosis.*—Chronic cardiac rheumatism with auricular fibrillation.

*History.*—One month previous to admission the patient developed bronchitis and remained in bed for a fortnight. She then got up for three days, but was compelled to return to bed again, her cough being very troublesome. A severe attack of breathlessness and palpitations followed. During this attack she vomited once.

*Previous history.*—Four attacks of rheumatic fever. During the last seven years she had suffered from heart trouble, and had frequently complained of pains round the heart.

*Condition on admission :—*

*Temperature*, 97·6° F. *Pulse rate*, 72. *Respirations*, 24 per minute. The patient looked ill and pale, with slight *cyanosis* of the cheeks. The *posture* assumed was semi-upright. Considerable dyspnœa present. *Tongue* clean but dry. Slight *œdema* of the legs and lumbar region of the back. *Sleep* rather intermittent, poor. *Bowels* opened fairly regularly. *Micturition*, normal.

*Pulse* feeble, very irregular in force and frequency. *Blood pressure*, systolic 130, diastolic 70. *Apex beat* in left sixth space four inches from the mid-line. Very diffuse.

*Area of cardiac dullness*, left border of the sternum, four inches from the mid-line in the sixth left space ; in the third left rib above.

*Heart sounds* extremely irregular. A systolic murmur was discernible, however, and was loudest at the apex, but was also faintly heard at the base over the aortic area. This systolic murmur was conducted out to the axilla.

Percussion note was found to be slightly impaired at the right base. Vocal fremitus, vocal resonance and breath sounds were normal.

*Abdomen.*—Area of liver dullness was found to extend down as far as two inches below the costal margin. No other abnormal signs were found.

*Nervous system*, normal.

*Urine.*—Specific gravity = 1008. Reaction, acid. No blood, albumen or sugar present.

A fortnight after admission the patient showed greater cyanosis. Dyspnœa was very severe at times. She had assumed an almost completely upright sitting posture. *An œdema of the left arm* had developed, and the veins over the left side of her chest were very dilated ; the right side was normal. During the last three weeks of life the patient became even more dyspnœic, and she vomited several times.

*Ventricular failure* usually occurs as the result of high blood pressure or aortic incompetence, syphilis being a common cause. This is a more serious condition of affairs than signs of heart failure due to a bacterial intoxication as in diphtheria. In this latter condition there is always the possibility that with efficient nursing the patient may recover. In the former, however, death is the inevitable result, as the condition is gradually, if not rapidly, progressive. This type of failure is characterised by dyspnœa and cyanosis, which is chiefly due to right ventricular failure, and is seen in one of its deepest forms in pulmonary atheroma. Œdema is another early sign. Pain is due to left ventricular failure. This subject has been previously discussed, except for the pain due to coronary thrombosis. This takes the form of a sudden attack of pain in the chest and epigastrium, commencing in the night. It lasts for several hours, but gradually dies away, and the patient partially recovers. A few days later, however, a second attack occurs with a fatal result.

The blood pressure taken by means of a sphygmomanometer is often useful in detecting ventricular failure. Some useful signs obtained by it are :—

(*a*) A low systolic pressure in comparison to the diastolic, particularly significant when the systolic pressure has fallen rapidly.

(*b*) Alternation of the pulse can be detected if present. This is called "pulsus alternans," and is due to inefficiency of the left ventricle.

Certain signs of ventricular failure can be obtained by examination of the chest. They consist in a displacement of the apex beat outwards and to the left, and a rapid increase in the area of cardiac dullness. Faint heart sounds and tripling of the heart sounds (*i.e.* when a third sound is heard between the first and second sounds) may indicate ventricular failure.

**Case 2.**—H. S., female, aged 59. Married. Admitted to Bristol General Hospital on September 23rd, 1925.

*Diagnosis.*—Chronic cardiac rheumatism with aortic valvular disease, in which auricular fibrillation had occurred.

*History.*—Patient complained of severe breathlessness for twelve months previous to admission, worse at night than in the day. This had recently increased in severity. For several months she had noticed an increasing distension of her abdomen, and for four months had complained of swelling of her legs.

*Previous history.*—Rheumatic fever twenty-five years previously.

*Condition on admission* :—

*Temperature*, 96·5° F. *Pulse*, 88. *Respiration*, 28 per minute. *Posture*, semi-upright. Deep *cyanosis* and severe *dyspnœa*. Considerable *œdema* of back and legs. *Sleep*, very poor. *Bowels* opened regularly. *Micturition* normal.

*Pulse*, irregular in force and frequency. Tension fairly high. *Supra-sternal pulsation* present with a well-marked thrill. *Apex beat* in fifth left space, five and a half inches from the mid-line.

*Area of cardiac dullness*, third left rib above ; right sternal border to five and a half inches from the mid-line in fifth left space. There was also an area of dullness over the manubrium.

*Heart sounds*, irregular. At the *apex*, first sound replaced by a long systolic murmur. Second sound very faint. At the *base*, in the second right space (*i.e.* the aortic area), first sound replaced by a long systolic murmur. Second sound faint. At the second left space (*i.e.* at the pulmonary area) first sound was replaced by a long systolic murmur. Second sound slightly accentuated. A diastolic murmur was heard in the fourth left space near the sternum.

*Lungs*, normal.

*Abdomen.*—Distended and tense. Fluid thrill felt. Liver palpable, extending down about half an inch below the right costal margin.

*Nervous system*, normal. Wassermann reaction negative.

September 26th.—Abdomen soft and flabby. Shifting dullness but no fluid thrill. *Blood pressure*, right arm, systolic 135, diastolic 105 ; left arm, systolic 135, diastolic 115.

October 10th.—The apex beat of the heart was four inches from the mid-line.

*Area of cardiac dullness*, third left rib above ; mid-sternal line to four inches external to the mid-line in fifth left space. The liver had decreased in size considerably.

November 21st.—The rhythm of the heart was regular. Loud systolic and soft diastolic murmurs present at the apex. Systolic and diastolic murmurs present over aortic area. The diastolic murmur could be traced down the left border of the sternum to the apex, whilst the systolic could be traced up into the carotids.

The patient was discharged on November 24th.

*Auriculo-ventricular failure.*—This results from disturbance of the conducting system. Arterial lesions and gumma are the common causes. It may be manifested in three degrees :—

(1) The *a–c* interval in the jugular pulse may be lengthened.

(2) Ventricular beats may be dropped owing to occasional blocking of the passage of the stimulus from auricle to ventricle.

(3) A condition of heart block may exist when the auricles beat at twice or even four times the rate of the ventricle, or in cases of complete block the auricles and ventricles each beat at their own speeds.

**Case 3.**—G. P., male, age 51. Occupation, fish merchant. The patient gave a history of Adams-Stokes syndrome. He complained of having had attacks of fainting for four years. Sometimes the attacks occurred every few minutes and continued for several weeks, after which a period free from attacks followed. He also complained of a tightness at times in his chest but no pain.

*History of attack.*—Patient suddenly caught his breath, and lost consciousness for a few seconds. Extreme pallor was followed by excessive flushing as consciousness returned. Afterwards he suffered from headache.

*Previous history.*—(*a*) Rheumatic fever three times; (*b*) Frequent pains in joints.

*Condition on examination.*—Patient was somewhat pale. His movements were slow. Attitude, stooping. No dyspnœa and no œdema. Marked pulsation on both sides of the neck.

*Cardio-vascular system.—Pulse*, 64 per minute, slightly irregular in force and frequency. Tension good.

*Blood pressure*, systolic 160, diastolic 82.

*Cardiac impulse* visible to the left of the nipple.

*Apex beat* palpable five and a half inches from mid-line in the fifth left space.

*Area of cardiac dullness* (mapped out by percussion with some difficulty, as the patient was very muscular), from third left rib above; left sternal border to one inch outside the nipple line in fifth left space.

*Heart sounds*, very faint. At *the apex* both sounds were faintly audible. A murmur of the nature of a pericardial rub was heard in the fifth space over an area of two square inches extending from the nipple line towards the axilla. It was systolic in time. At *the base* a similar murmur was heard over the pulmonary area, and it masked the first pulmonary sound. The second pulmonary sound was slightly accentuated. Over the aortic area the first sound was not heard, the second sound was faint.

*Lungs and abdomen*, normal.

*Nervous system*, knee-jerks active. No other abnormal signs present.

*X-ray examination* of the chest by screening the patient showed the heart to be greatly enlarged, all the enlargement being left-sided. The greatest horizontal measurement was eight inches. The ratio of the greatest width of the heart to the width of the chest at that level was calculated to be 1 : 1·7.

*Electro-cardiogram (taken on the same day that the general examination was made).*

(1) Heart rate, 66 per minute.

(2) Leads I, II and III show delayed conduction of the auriculo-ventricular bundle, the P—R interval being more than 4/25 second. It gradually increases frcm 9/25 to 13/25 seconds, and occasionally conduction fails. Lead III therefore shows a dropped beat.

(3) Q R S shows notching in Lead I.

(5) Lead I—P is probabiy buried in T. Lead III—P is inverted.

Atropine was administered to this patient with excellent results on several occasions. This proves that the vagus was an important factor in producing the depression of conduction.

I have to thank Dr. Odery Symes, Dr. Carey Coombs and Dr. Carleton for permission to make use of their cases.

REFERENCE.

[1] *Medical Science; Abstracts and Reviews,* Nov., 1924: Abstract on Dyspnœa by J. H. Means.

## Figures and Tables

**Figure f1:**